# Comparison of computer-aided and manual measurements in the evaluation of carpal alignment

**DOI:** 10.1177/17531934231220637

**Published:** 2023-12-16

**Authors:** Robert Sippo, Kira Huuska, Theresa Höglund, Eero Waris

**Affiliations:** 1 Department of Hand Surgery, Helsinki University Hospital and University of Helsinki, Helsinki, Finland.; 2 Mehiläinen Helsinki Hospital, Helsinki, Finland

**Keywords:** Carpal alignment, measurement, reliability, computer-aided, digitally reconstructed radiographs

## Abstract

The purpose of this study was to compare computer-aided analysis and different methods of manual measurements in the evaluation of carpal alignment. The radioscaphoid, radiolunate, radiocapitate and radiometacarpal angles were measured on cone-beam computed tomography (CT) scans of 30 healthy wrists by automated software (Disior Ltd.) and by hand surgeons using lateral radiographs reconstructed from the CT data. Hand surgeons were either given (*n* = 6) or not given (*n* = 7) prior instructions on how to perform the measurements. Inter- and intra-observer reliability of manual measurements ranged from good to excellent (intra-class correlation coefficients [ICC] 0.77–0.99), being highest in specialists with standardized methods and in reconstructed radiographs with bone overlap digitally removed. Computer-aided software provided excellent intra-observer reliability (ICC 0.94–1.00) consistently and values that were highly comparable (mean difference range 1°–7°) with the manual measurements made in optimal settings. Computer-aided software provides an accurate and repeatable method to measure carpal alignment in CT scans, minimizing observational errors.

## Introduction

Carpal alignment measurements are utilized in diagnosing carpal instabilities. The alignment is currently assessed in lateral radiographs by tracing carpal bone axes and measuring the angles between them (Linscheid et al., 1972). These measurements are prone to various errors. The visual identification of bony landmarks can be difficult due to overlapping structures (Garcia-Elias et al., 1989). There are varying definitions for the axis landmarks (Larsen et al., 1991) and consensus regarding the optimal method has not been reached. Furthermore, variation in the projections and positioning of the upper extremity can lead to differences in measured values (Capo et al., 2009; Koh et al., 2013). Thus, the reliability of manual measurements has been reported to be variable (Cho et al., 2006; Garcia-Elias et al., 1989; Larsen et al., 1991; Nakamura et al., 1989; Roh et al., 2014). In optimized settings with instructions and guides to facilitate the manual measurements, high inter- and intra-observer reliabilities can be reached (Ferreira Branco et al., 2022; Lee et al., 2018). However, this may not represent the clinical reality of hand surgeons conducting carpal alignment measurements with individual variability (Suojärvi et al., 2021a).

Computed tomography (CT) is increasingly used in wrist imaging (Gibney et al., 2019; Neubauer et al., 2016; Pallaver and Honigmann, 2019). Cone-beam technology, with a relatively low radiation dose and high spatial resolution, allows free positioning of the wrist and arm (Koivisto et al., 2018). Although visualization of individual carpal bones is easier in CT images, bony landmarks are often visible on different slices of the image, which complicates measurements (Maizlin and Vos, 2012; Tan et al., 2014). CT data allow producing digitally reconstructed radiographs to enable comparative measurements with values originally defined in plain radiographs (Robertson et al., 2002; Schormans et al., 2019). Recently, a computer-aided CT analysis technique based on segmentation and mathematical modelling to allow automated measurement of carpal alignment on wrist cone-beam CT images was introduced (Höglund et al., 2023; Sippo et al., 2021).

The purpose of this study was to compare computer-aided software and manual measurements in the evaluation of carpal angles in neutrally positioned, asymptomatic wrist cone-beam CT scans. We assessed the inter- and intra-observer reliability of manual measurements using digitally reconstructed lateral radiographs created from the same cone-beam CT data analysed by the software. The manual measurements were performed by hand surgeons without prior instructions on reconstructed summation lateral radiographs to simulate clinical practice. In addition, to standardize the measurement methods and to minimize observational errors in the manual measurements, a second set of hand surgeons who were instructed how to draw the carpal axis variants were also asked to draw the axes on stripped reconstructed lateral radiographs without bone overlapping. These optimized measurements were compared with the angle values measured by the software to investigate its validity in carpal alignment assessment.

## Materials and methods

### Study population

The study was approved by the ethics committee of Helsinki University Central Hospital and the institutional review board. A total of 30 wrist CT scans of asymptomatic volunteers (15 men, 15 women; mean age 38 years [SD 9.5], range 22–60) with no history of wrist trauma, malformations or wrist pain, acquired in the neutral position with a cone-beam CT scanner (Planmed Verity, Planmed Oy Helsinki Finland) were used for this study. These 30 scans were a subset of an original cohort of 121 wrists with the same inclusion criteria imaged for and analysed in a previous study, which focused on carpal bone axis definitions and normal values in asymptomatic wrists (Sippo et al., 2021). Of the wrists, 20 were imaged twice to enable intra-observer reliability assessment by the analysis software. Ten additional wrists were randomly chosen from the original cohort for sufficient power for the statistical analysis. All participants submitted written consent before imaging. Image data were pseudonymized and exported.

### Software measurements

A software (Disior Oy, Helsinki Finland) based on segmentation of CT images and mathematical modelling (Suojärvi et al., 2021b) was utilized. In the software, the axis of the radius was defined using a segment 28.8 mm and 53.3 mm from the distal articular surface (Suojärvi et al., 2021b). For the middle metacarpal, an axial axis was defined using a 10 mm segment of the proximal metaphysis/diaphysis. For the scaphoid and capitate, an axial axis was defined by plotting a line through the geometric centre points of 0.5 mm slices along the bone’s length. For the lunate, an axis perpendicular to the distal articular surface was used as analogous to the lateral radiograph axial axis drawn by the observers. A palmar tangential axis was defined for the scaphoid and a dorsal tangential axis for the middle metacarpal and capitate. For the lunate, a tangential axis was defined as a line perpendicular to the line connecting the tips of the distal horns. The axes were projected on to a lateral plane defined by the scaphopisocapitate (SPC) alignment (Yang et al., 1997). The software then calculated the radiocarpal angles, which were used as the basis for intra-observer reliability calculation for the software, and these were compared to the angles calculated from the human observers’ annotations. Radio-middle metacarpal, radioscaphoid, radiolunate and radiocapitate angles were assessed.

### Observers

Two groups of observers were recruited. The first group included seven specialist hand surgeons with a mean experience of 17 years (SD 11, range 6–41) after specialist degree. This group received no prior instructions or information about the task nor guidelines for determining the axes. The second group included six specialist hand surgeons with a mean experience of 8 years (SD 5, range 3–17 years) after specialist degree. This group was provided with a written and illustrated instruction guide for the measurements (Supplementary Figure S1).

### Images

**Figure 1.**
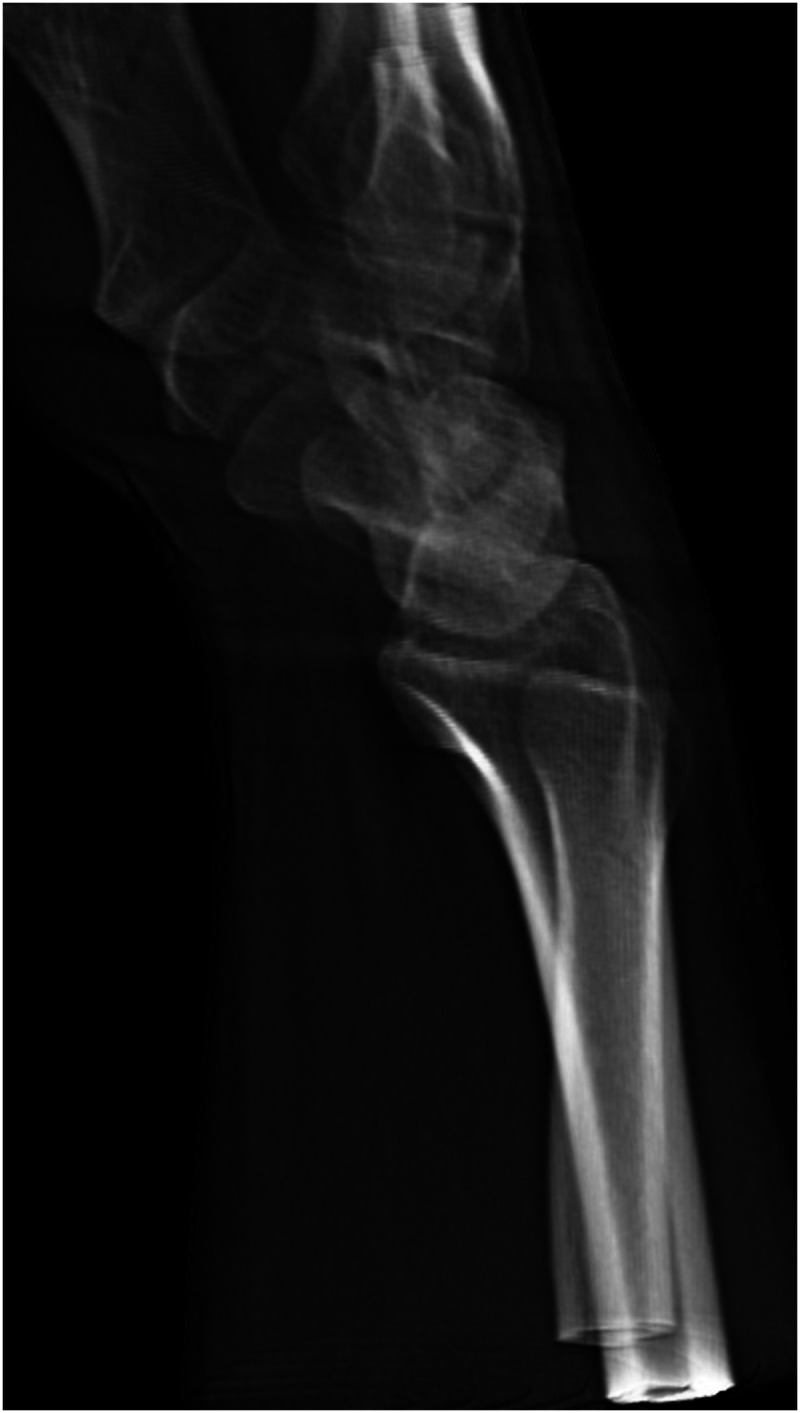
A summation radiograph reconstructed from cone-beam CT data.

**Figure 2.**
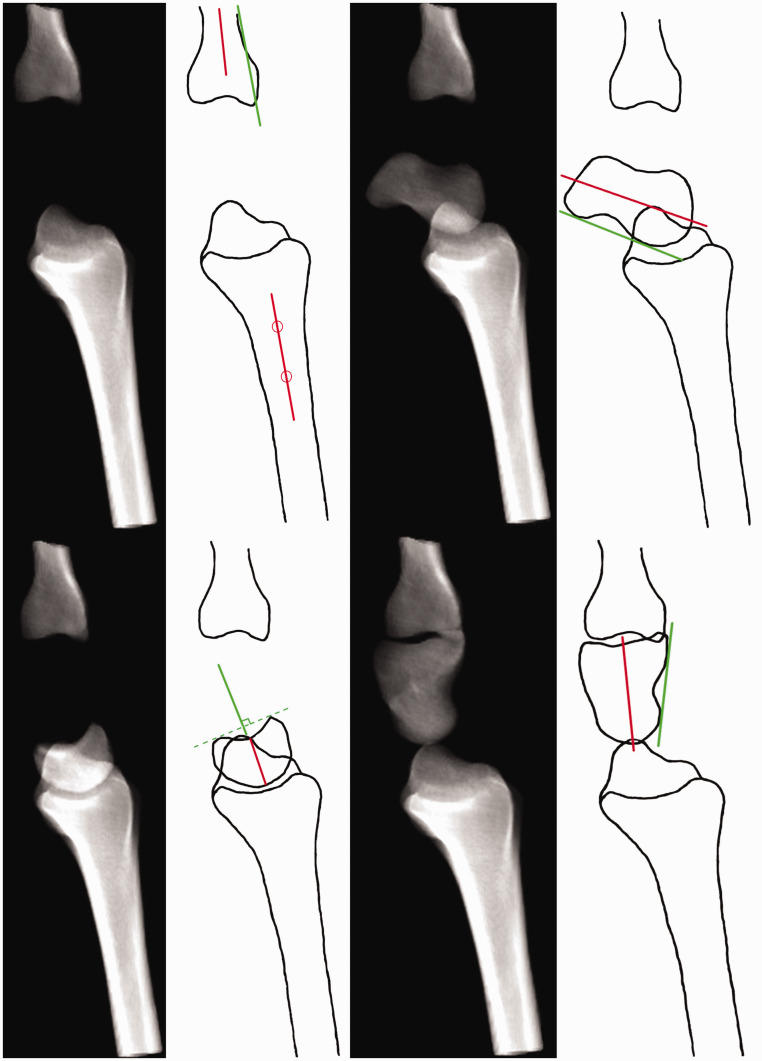
Reconstruction images with bone overlap removed, with schematic representations of the axial (red) and tangential (green) axes.

The CT image data of each wrist were digitally reconstructed into a two-dimensional summation image containing all the imaged bones mimicking a lateral radiograph, fulfilling the SPC relationship criteria (Yang et al., 1997) (Figure 1). To minimize landmark obstruction, a second reconstruction image set was created similarly while digitally removing bones not being assessed, for example, the scaphoid axes were marked on an image showing only the scaphoid in an otherwise empty carpus with the radius and middle metacarpal as a frame of reference (Figure 2). All images were first aligned with the axis of the distal radius as vertical and then pseudorandomly rotated 1°–10° in the sagittal plane. The same rotation was maintained for all reconstructed images of a particular wrist. The amount of rotation was noted and used to assess the error in measuring the axis of the radius by calculating the angle difference between the drawn axis, the vertical axis of the whole image in the picture archiving and communication system (PACS) viewer drawn as a pixel-perfect vertical line from the top of the screen to the bottom, and the known rotation.

### Observer measurements

The images were analysed in the PACS software (Syngo.plaza, Siemens Healthcare GmbH, Erlangen Germany) using standard workstation monitors. The first observer group was asked to draw the axes for the radius, scaphoid, lunate, capitate and the middle metacarpal using axis definition they preferred in their daily practice The first group only annotated the summation images. The second group was given axes definitions and was instructed to draw the axis of the radius and two alternate axes for the rest of the bones on both the summation and stripped images (Table 1). The images were viewed in a randomized order. The annotations were repeated after a minimum of 1 month in a freshly randomized order to negate memory bias. The annotated images were exported in Digital Imaging and Communications in Medicine (DICOM) format and analysed by the lead authors. The images were windowed to black to only show the drawn lines and thus remove any bias or disagreement of the visible landmarks. The angles between the drawn axes and the vertical axis of the image were meticulously measured. These measurements were used in the statistical analysis.

**Table 1.** Axis definitions for the instructed human observers.

Axis	Axis definition	
	Axial	Tangential
Radius	A line connecting points in the middle of the medullary canal 3 cm and 5 cm proximal of the lunate fossa	
Middle metacarpal	A line along the middle of the medullary canal	A tangent of the dorsal margin of the diaphysis
Scaphoid	A line connecting the midpoints of the proximal and distal poles	A tangent of the proximal and distal palmar margins
Lunate	A line connecting the midpoints of the proximal and distal articular surface	A line perpendicular to the line connecting the distal tips of the articular concavity
Capitate	A line connecting the midpoints of the proximal and distal poles	A tangent of the proximal and distal dorsal margins

### Statistical analysis

Intra-class correlation coefficients (ICC2,1) were used to measure the intra- and inter-observer reliability. ICC values are in the range of 0–1, where 1 indicates absolute agreement among observers and an ICC value of 0 indicates total disagreement. Poor reliability was defined as <0.5, moderate reliability as 0.5–0.75, good reliability as 0.76–0.9 and excellent reliability as >0.9 (Koo and Li, 2016). The intra-observer ICC for the software analysis was calculated using the 20 twice-imaged wrists by comparing the measurements acquired in the first and second respective scans. Intra-observer ICC for the software’s radius axis determination was calculated by comparing the amount of rotation in degrees needed to align the axis with vertical axis in the twice-imaged wrists. The 20 wrists used for the ICC calculation using software were included in the 30 measured by the observers. Intra-observer ICC for the observers was calculated for each axis by comparing the first and second measurement rounds. Inter-observer ICC was calculated by comparing the observer measurements of the first round against each other. The uninstructed and instructed groups were calculated separately.

Radiocarpal angles calculated from the observer measurements in the stripped images and the angles calculated by the software were compared using the Student’s *t*-test. A Bland–Altman analysis was performed to evaluate the agreement between observer and software observations. Angles between axial and tangential axes were compared using the Student’s *t*-test. Correlation with observer experience in years with the intra-observer ICC was assessed with the Spearman’s correlation test. A *p*-value <0.05 was considered significant.

## Results

The results of the ICC analysis are shown in Table 2. Overall, the intra- and inter-observer reliability of the manual measurements ranged from good to excellent. The axes of the radius and the middle metacarpal were identified very consistently (ICC values in the range of 0.91–0.98). The carpal bone axes produced more variation. The inter-observer reliability improved from the uninstructed group to the instructed group in the summation radiographs and further improved in the stripped radiographs. The intra-observer reliability was similar with and without instructions in the summation radiographs and improved to a nearly perfect agreement in the stripped images. The intra-observer reliability of the computer-aided analysis was similar to the observer inter- and intra-observer reliability in the stripped radiographs, demonstrating nearly perfect agreement (ICC values in the range of 0.94–1.00).

**Table 2.** Intra-class correlation coefficients.

	Observers without instructions		Observers with instructions				
	Summation image ICC		Summation image ICC		Stripped image ICC		Software ICC
Axes	Inter-observer	Intra-observer	Inter-observer	Intra-observer	Inter-observer	Intra-observer	Intra-observer
Radius	0.97	0.99 (0.01; 0.98–0.99)	0.94	0.96 (0.03; 0.89–0.99)	0.97	0.98 (0.01; 0.95–0.99)	1.00
Middle metacarpal	0.91	0.93 (0.04; 0.85–0.96)					
Axial			0.94	0.95 (0.02; 0.91–0.97)	0.96	0.93 (0.12; 0.66–0.99)	0.99
Tangential			0.96	0.96 (0.01; 0.95–0.99)	0.98	0.99 (0.01; 0.97–1.00)	0.97
Scaphoid	0.77	0.9 (0.03; 0.84–0.92)					
Axial			0.84	0.90 (0.04; 0.83–0.94)	0.95	0.97 (0.01; 0.94–0.98)	1.00
Tangential			0.91	0.92 (0.04; 0.88–0.99)	0.99	0.99 (0.002; 0.99–0.994)	1.00
Lunate	0.85	0.89 (0.07; 0.73–0.96)					
Axial			0.87	0.90 (0.05; 0.80–0.95)	0.93	0.96 (0.02; 0.92–0.98)	0.98
Tangential			0.93	0.91(0.03; 0.87–0.97)	0.99	0.99 (0.01; 0.96–1.00)	0.94
Capitate	0.84	0.85 (0.07; 0.70–0.94)					
Axial			0.85	0.91 (0.03; 0.85–0.94)	0.96	0.97 (0.01; 0.95–0.99)	0.99
Tangential			0.85	0.87 (0.06; 0.74–0.90)	0.98	0.99 (0.01; 0.98–1.00)	1.00

Data are expressed as mean (SD; range)

ICC: intra-class correlation coefficient.

**Figure 3.**
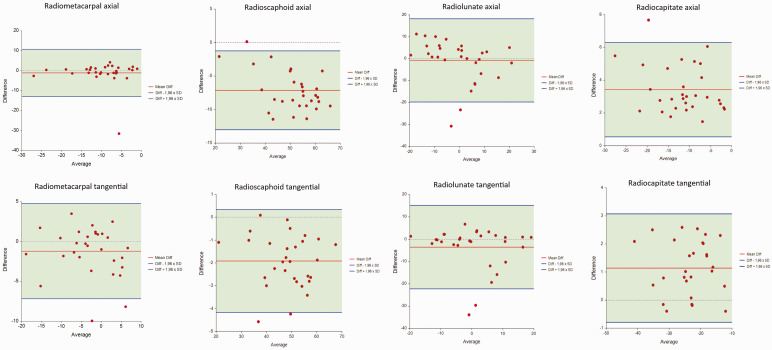
Bland–Altman graphs comparing the observer mean and software measurements.

The differences between the measurements performed by software and the observers using digitally reconstructed radiographs are presented in Table 3. The greatest difference was in the axial radioscaphoid angle with a mean difference of 7°. The other axes produced mean differences of 1°–3°. Ranges and standard deviations of the measured angles were similar. The Bland–Altman graphs showed a slight increase in difference in the axial scaphoid axis for greater radioscaphoid angles (the scaphoid more in flexion) and in the axial lunate axis for lesser radiolunate angles (the lunate more in extension) (Figure 3). The rest of the Bland–Altman graphs showed a scattershot pattern of no bias in the measurement differences. The differences were mostly within 2 SD of the mean difference for each angle. The Bland–Altman graphs also showed two outliers in the lunate measurements and one in the axial radiometacarpal measurements with the observer mean and software measurement difference over 20°, due to a segmentation error by the software leading to abnormal measurement values.

**Table 3.** Comparison of measured angles (in degrees); observers and axis variants.

Angle	Observers	Software	*p*-value
Radiometacarpal			
Axial	−10 (7; −29–4)	−9 (7; −26–10)	**0.03**
Tangential	−3 (7; −23–8)	−2 (7; −18–10)	**0.02**
*p*-value	**<0.001**	**<0.001**	
Radioscaphoid			
Axial	47 (10; 15–67)	54 (11; 23–71)	**<0.001**
Tangential	47 (10; 18–69)	49 (10; 22–68)	**0.01**
*p*-value	0.90	**0.01**	
Radiolunate			
Axial	−1 (11; −22–29)	0 (13; −23–22)	0.32
Tangential	−1 (10; −22–22)	2 (11; −20–19)	**<0.001**
*p*-value	0.73	0.30	
Radiocapitate			
Axial	−10 (6; −27–3)	−13 (7; −30–−3)	**<0.001**
Tangential	−23 (7; −41–−4)	−24 (7; −42–−12)	**0.02**
*p*-value	**<0.001**	**<0.001**	

Data are expressed as mean (SD; range). Significant *p*-values shown in bold. The middle metacarpal was chosen for the radiometacarpal angle.

In the instructed observer group, the intra- and inter-observer ICC were higher for the tangential axes than the axial axes both in the summation and stripped images. In the uninstructed observed group, all seven observers opted to draw the axial axis for all carpal bones excluding one who drew tangential axes for the scaphoid and lunate. Table 3 also shows the difference in angles produced by axial and tangential methods. The axial axis produced lesser radiometacarpal angles than the tangential axis. The tangential axis produced lesser radiocapitate angles than the axial axis. Both differences were similar in observer and software measurements. There was no correlation between observer experience and intra-observer ICC in either observer group (*p*-values 0.354–0.658).

## Discussion

This study shows that computer-aided analysis offers an accurate and reliable method to measure carpal alignment. The intra-observer reliability of the software analysis was excellent and on par with the highest ICC values obtained by the hand surgeons measuring digitally reconstructed radiographs in optimized settings with standardized methods and landmark obstruction removed. Furthermore, the carpal angles measured by the software were closely comparable to the manual measurements. The use of digitally reconstructed radiographs from CT image data allowed direct comparison between manual and software measurements. Although statistically significant differences were noted, most of the differences were in the range of 1°–3°, too small to be clinically significant. The largest difference, mean 7° greater angle values measured by the software, was seen in the scaphoid axial axis values, which may result from the three-dimensional geometric scaphoid axis used by the software being projected on to a two-dimensional sagittal plane. In the Bland–Altman analysis of the axial scaphoid axis, the difference was slightly greater in greater radioscaphoid angles, suggesting human observers may tend to underestimate the flexion of the scaphoid. No consistent difference has been found between normal values for the population calculated using computer-aided analysis reported earlier and the normal values measured manually in standard radiographs reported in previous published studies (Sippo et al., 2021).

In our study, the intra- and inter-observer reliability of observers with instructions ranged from good to excellent. Most previous studies on radiocarpal and intercarpal measurement reliability have used experienced observers with dedicated instructions, with varying results. Cho et al. (2006) had 20 observers to assess five radiographs for radiolunate, scapholunate and lunocapitate angles, reaching intra- and inter-observer reliability ranging from poor to moderate (ICC values of 0.04–0.70). Roh et al. (2014) had three observers measure radiocarpal and intercarpal angles in 36 radiographs with scaphoid fractures and reached mostly good intra- and inter-observer agreement (ICC values of 0.57–0.85). Our results were in agreement with Lee et al. (2018) who had three observers to assess 30 wrists imaged with radiographs and CT. For the radiographs, intra- and inter-observer reliability ranged from good to excellent (ICC values of 0.86–0.98). In our study, digitally removing landmark overlap improved reliability to a near perfect agreement. Lee et al. (2018) also assessed CT images reconstructed to show only the measured bones as a three-dimensional model and their observer agreement was excellent (ICC values of 0.94–0.99), which was very similar to our results. Similar very high reliability has recently been reported by Ferreira Branco et al. (2022) measuring radiocarpal angles in cone-beam CT images. Thus, in optimized conditions manual measurements can be reliable and repeatable. In clinical practice, however, physicians conduct carpal alignment measurement with individual variability produced by personal expertise and varying conditions. To simulate clinical reality, the manual measurements in our study were also performed on digitally reconstructed lateral radiographs by hand surgeons without prior instructions. In this group, the carpal bone axes, particularly in the scaphoid, were found less reliably. Previous studies suggest there need to be a difference of at least 5°–8° in carpal angles for it to be detectable by human observers (Garcia-Elias et al., 1989; Larsen et al., 1991). In our data, a measurement difference of 5° between measurement sessions produced an intra-observer ICC of 0.86. Thus, the uninstructed expert observers were at or below the threshold of detection for carpal angle abnormalities. It is probable that carpal angle measurement reliability of less experienced observers in a clinical setting is even lower. Suojärvi et al. (2021a) noted poor to good inter-observer reliability with uninstructed observers measuring distal radius parameters, lower than previous distal radius studies with instructed observers. The effect of instructing observers has, to our knowledge, not been studied before with regards to carpal angle measurements.

Standardization of measurement techniques may enable more consistent measurement of the carpal alignment. In our study, higher inter- and intra-observer ICCs were obtained when using tangential axes compared to axial axes. Garcia-Elias et al. (1989) found no difference in reliability between axial and tangential axes, while Larsen et al. (1991) found the tangential axes to be more reliable, most likely because the outlines of the bones are less occluded by overlap. In clinical practice there is variation to which axes are used, as evident by our uninstructed observers electing to use the axial axes more often. We recommend using the dorsal tangential axis for the middle metacarpal, the palmar tangential axis for the scaphoid, the perpendicular to distal tips line axis for the lunate and the dorsal tangential axis for the capitate when manually measuring carpal alignment. The differences between the angles produced by different axis variants should be noted when interpreting results (Table 3).

The present study has some limitations. These include the use of only cone-beam CT images of asymptomatic wrists, using the scanner of a single manufacturer, and the use of digitally reconstructed radiographs in the manual measurements. While the digital reconstruction of the CT data allowed direct comparative analysis of measurements and ensured identical wrist position and optimal projection, the reconstruction process may highlight or diminish some details in the images compared to the traditional radiographs. Further studies are needed to confirm the reliability of computer-aided analysis in wrists with carpal instability, and to test the performance of the software with CT and MRI scanners of different manufacturers.

In this study, computer-aided cone-beam CT image analysis software was validated over a cohort of asymptomatic wrists. The software has the potential to provide an automated assessment of carpal alignment with high reliability and accuracy, comparable to that of expert hand surgeons in optimal settings. The segmentation and numerical modelling of CT data also allows quantification of three-dimensional carpal alignment allowing more detailed alignment analysis (Höglund et al., 2023). If integrated into clinical practice, automated alignment interpretation may serve as an adjuvant to the physicians’ assessment, highlighting abnormal values and reducing time spent while minimizing diagnostic errors. It also allows serial image comparison, which may be useful in analysing progression of carpal instabilities and kinematics of the wrist. Further studies are underway to examine carpal alignment in wrists imaged in non-neutral positions and during motion, and in wrists with instabilities or degeneration.

## Supplemental Material

sj-pdf-1-jhs-10.1177_17531934231220637 - Supplemental material for Comparison of computer-aided and manual measurements in the evaluation of carpal alignment Supplemental material, sj-pdf-1-jhs-10.1177_17531934231220637 for Comparison of computer-aided and manual measurements in the evaluation of carpal alignment by Robert Sippo, Kira Huuska, Theresa Höglund and Eero Waris in Journal of Hand Surgery (European Volume)
