# Supplementary material for: Comparison of computer-aided and manual measurements in the evaluation of carpal alignment
Source: J Hand Surg Eur Vol. 2023 Dec 16;49(8):987–94. doi: 10.1177/17531934231220637 (PMC11382436; doi:10.1177/17531934231220637)
Supplement: sj-pdf-1-jhs-10.1177_17531934231220637 - Supplemental material for Comparison of computer-aided and manual measurements in the evaluation of carpal alignment [file sj-pdf-1-jhs-10.1177_17531934231220637.pdf]

Carpal alignment measurements

Image 1:

Draw the axis of the radius

- a line connecting two points in the middle of the medullary canal 3cm and 5cm proximal from the articular surface

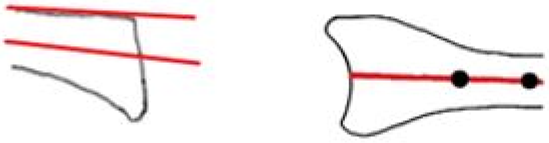

Draw the middle metacarpal axes

- A line tangential to the dorsum of the metacarpal
- A line along the medullary canal

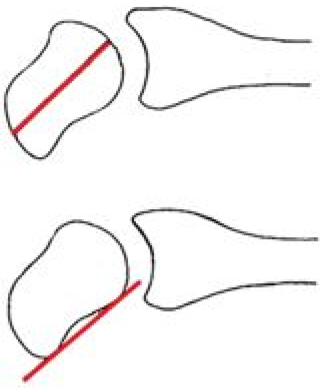

Image 2:

Draw the scaphoid axes

- A line connecting the midpoints of the proximal and distal poles
- A tangent of the proximal and distal volar margins

Image 3:

Draw the lunate axes

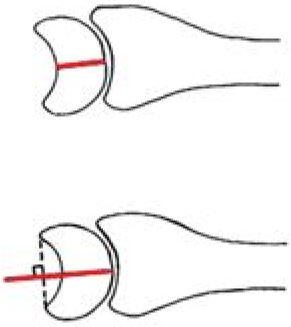

- A line connecting the midpoints of the proximal and distal articular surface
- A line perpendicular to the line connecting the distal tips of the articular concavity

Image 4:

Draw the capitate axes

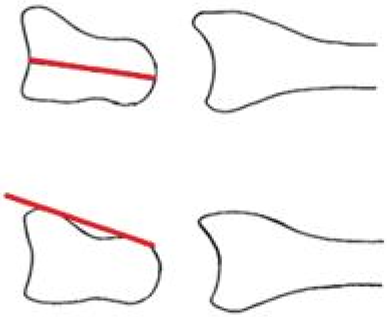

- A line connecting the midpoints of the proximal and distal poles
- A tangent of the proximal and distal dorsal margins

Summation Image:

Draw all the axes
